# Rapid Detection of Duck Enteritis Virus with MIRA, MIRA–qPCR, and MIRA–LFD Assays

**DOI:** 10.3390/pathogens14100980

**Published:** 2025-09-27

**Authors:** Yin Dai, Xiaomiao Hu, Yueyi Zhong, Liyuan Chen, Jieru Wang, Dongdong Yin, Lei Yin, Xuehuai Shen, Xiaocheng Pan, Xuelan Liu, Ruihong Zhao

**Affiliations:** 1 Anhui Provincial Key Laboratory of Livestock and Poultry Product Safety, Livestock and Poultry Epidemic Diseases Research Center of Anhui Province, Institute of Animal Husbandry and Veterinary Science, Anhui Academy of Agricultural Science, Hefei 230031, China; daiyin2020@163.com (Y.D.); huxiaomiao66@163.com (X.H.);; 2 College of Veterinary Medicine, Anhui Agricultural University, Hefei 230036, China

**Keywords:** duck enteritis virus, multienzyme isothermal rapid amplification, specificity, sensitivity, visible detection

## Abstract

Duck viral enteritis (DVE) is an acute and highly contagious disease that affects waterfowl such as ducks, geese and swans. Duck enteritis virus (DEV) is the pathogen, causing huge economic losses to waterfowl farming in recent years. Establishing a rapid, simple, and visual detection should facilitate the early identification of DEV. After the amplification primers and reaction conditions were optimized, three multienzyme isothermal rapid amplification (MIRA) methods—basic MIRA, MIRA–quantitative PCR (MIRA–qPCR) and MIRA–lateral flow dipstick (MIRA–LFD)—were established to detect DEV. Specificity analyses showed that the three MIRA methods specifically detected DEV, with no cross-reaction with fowl adenovirus serotype 4, novel goose astrovirus, Muscovy duck reovirus, avian influenza virus subtype H9, or duck circovirus. The basic MIRA reaction was completed in 30 min at 35 °C, requiring only a pair of primers. Detection with MIRA–qPCR or MIRA–LFD was completed within 20 min, and the limits of detection were 1 × 10^1^ copies/μL for both. MIRA–LFD required no specialized instruments, and the results could be viewed directly with the naked eye. Compared with the traditional PCR, MIRA assays are simple, rapid, and effective and therefore more suitable for the field detection of DEV.

## 1. Introduction

Duck viral enteritis (DVE), also known as duck plague, is an acute and highly contagious disease caused by duck enteritis virus (DEV) [1]. DEV belongs to the family *Herpesviridae* and sub-family *Alpha-herpesvirinae*, a double-stranded DNA virus [2,3]. DEV is the main pathogen of waterfowl such as ducks, geese and swans. This virus is widely distributed around the world and can cause a mortality rate of up to 100% in sick ducks, posing a significant threat to the waterfowl industry [4]. The transmission of DEV includes both horizontal and vertical ways, and migratory waterfowl play an important role in the spread of the virus [5]. In recent years, there have been a large number of waterfowl farms in central and southern China, and duck plague outbreaks have occurred frequently [6]. The adult duck disease caused by the highly pathogenic DEV-JS2024 strain in a breeding farm has caused serious economic losses [7].

Timely diagnosis and monitoring are essential for the prevention and control of DEV. Molecular diagnostic techniques have gained attention in the field of animal pathogen detection due to their high specificity and sensitivity. Among them, methods such as polymerase chain reaction (PCR), quantitative real-time PCR (qPCR), and loop-mediated isothermal amplification (LAMP) have been widely applied in DEV detection [8,9,10]. However, PCR and qPCR methods require sophisticated experimental equipment or skilled personnel, which limits their application in locations with limited resources. The design of LAMP primers is complex, requiring the creation of 4 to 6 specific primers, and the reaction temperature is relatively high, ranging from 50 to 65 °C [11]. To overcome these limitations, some new isothermal rapid amplification techniques have emerged as an effective alternative.

Multienzyme isothermal rapid amplification (MIRA) is a novel method of nucleic acid isothermal amplification. This approach relies on the synergistic action of four core functional proteins, namely recombinant enzyme, DNA helicase, single-chain antigen-binding protein, and DNA polymerase, to rapidly amplify target genes under isothermal conditions [12]. The basic MIRA method can also be combined with fluorescent quantitative PCR (MIRA–qPCR) or colloidal gold lateral flow dipstick (MIRA–LFD) to further improve the sensitivity and allow the visualization of the results [13,14]. At present, MIRA assay has not been applied to the detection of DEV. In this study, three rapid MIRA methods (MIRA, MIRA–qPCR and MIRA–LFD) were established for DEV.

## 2. Materials and Methods

### 2.1. Viruses and Plasmids

DEV, novel goose astrovirus (NGAstV), avian influenza virus subtype H9 (AIV H9), fowl adenovirus serotype 4 (FAdV-4), duck circovirus (DuCV), and Muscovy duck reovirus (MDRV) were maintained in our laboratory. The target fragment of thymidine kinase protein (TK) gene sequence was synthesized and was used to construct the recombinant pUC57–DEV plasmid by Sangon Biotech (Shanghai) Co., Ltd. (Shanghai, China).

### 2.2. Design and Synthesis of Primers and Probes

The primers and probes for the MIRA, MIRA–qPCR, and MIRA–LFD assays were designed based on the relatively conserved TK gene of an DEV strain (accession no. KF214788.1) in the GenBank database on 30 October 2023 (https://www.ncbi.nlm.nih.gov/genbank/). The sequences of primers and probes were designed and analyzed using PrimerSelect and MegAlign program of Lasergene 7.1 software package (DNAStar, Madison, WI, USA). In the probe of MIRA-qPCR, Fluorescein-deoxythymidine (FAM-dT) was the reporter group, and Black Hole Quencher-deoxythymidine (BHQ-dT) was the fluorescence quenching group. C3Spacer was a group that prevented the extension or amplification of polymerase. Tetrahydrofuran (THF) regulates the distance through conformational changes. They worked together to carry out the detection of target genes. FAM, THF and C3spacer were introduced into the probe of MIRA-LFD. In addition, biotin in the primer R1 acted as a “molecular bridge”, which had high specificity and signal amplification effect. The specific primer and probe sequences for DEV for the three MIRA methods were listed in Table 1. They were synthesized and purified by Sangong Biotechnology (Shanghai) Co., Ltd. (Shanghai, China).

### 2.3. Extraction of RNA and DNA

Total viral DNA and RNA were extracted with M5 HiPer Viral DNA/RNA Kit (Catalog number MF116-01), according to the manufacturer’s instructions. The viral RNA was then reverse-transcribed to complementary DNA (cDNA) using the M5 HiPer First Strand cDNA Synthesis Kit (Catalog number MF1129-01). Both commercial kits were purchased from Beijing Mei5 Biotechnology Co., Ltd. (Beijing, China).

### 2.4. Establishment of Basic MIRA, MIRA–qPCR and MIRA–LFD

The *DNA* basic *rapid amplification kit* (Catalog number WLB8201KIT), fluorescent *rapid amplification kit (Catalog number WLE8202KIT) and rapid amplification kit for colloidal gold strip (Catalog number WLN8203KIT)* were used for basic MIRA, MIRA–qPCR and MIRA–LFD, respectively. All three kits were purchased from Weifang Amp-Future Biotech (Shandong, China), and the experiments were carried out in accordance with the manufacturer’s instructions.

### 2.5. Optimum Temperature and Time for Basic MIRA Assay

To determine the optimal reaction conditions for basic MIRA detection, different temperatures (20–40 °C) and times (3–40 min) were used during the detection process. The test results were analyzed, and the better reaction temperature and time were selected.

### 2.6. Specificity and Sensitivity of Three MIRA Assays

The nucleic acids of DEV, NGAstV, FAdV-4, DuCV, AIV H9 and MDRV were used as the templates to determine the specificity of the MIRA, MIRA–qPCR, and MIRA–LFD assays. ddH_2_O was used as the template in the negative control for each test. The recombinant plasmid pUC57–DEV was serially diluted 10-fold with ddH_2_O to concentrations of 1 × 10^9^–1 × 10^1^ copies/μL. The sensitivity of the three MIRA methods was the lowest detectable concentration.

### 2.7. Assay Evaluation with Clinical Samples

The clinically diseased tissues from dead waterfowl (ducks and geese) were collected by technicians from poultry farms in Anhui Province of China and sent to the laboratory. In total, 40 clinical samples were used for the detection of DEV with a conventional PCR assay and the three MIRA assays (basic MIRA, MIRA–qPCR, and MIRA–LFD). The conventional PCR was performed as described in a previous report [15]. The detection numbers, detection rates, and coincidence rates of the PCR and the three MIRA methods were compared. The amplification products of the detected samples were sequenced by Sangong Biotechnology (Shanghai) Co., Ltd. (China). If they were consistent with the target gene sequence, they were confirmed as positive samples.

## 3. Results

### 3.1. Validation of Primers and Probes

**Figure 1:**
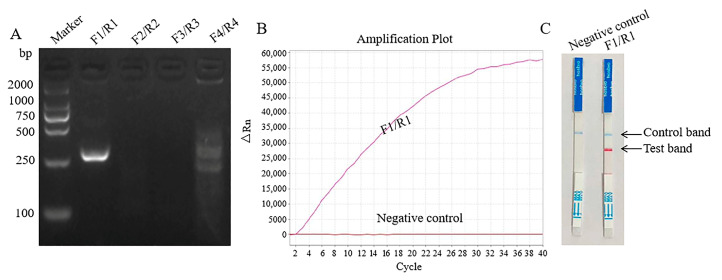
Primary tests for primers and probes of three MIRA assays. (**A**) Agarose gel electrophoresis of basic MIRA assay. (**B**) The amplification results of MIRA-qPCR assay. (**C**) Visual detection of MIRA-LFD assay.

The primers and probes were tested with the three MIRA assays using the recombinant plasmid pUC57–DEV with a concentration of 1 × 10^6^ copies/μL as the template. First, the four pairs of primers for the basic MIRA were tested, and only the first pair of primers had bright target bands (Figure 1A). MIRA relies on isothermal amplification, and the morphology of its amplification curve is different from that of traditional qPCR. As shown in Figure 1B, a clear amplification curve was produced in the MIRA–qPCR assay, and the target DEV gene was also cloned with the MIRA–LFD method (Figure 1C). All negative controls produced nonspecific amplification bands.

### 3.2. Optimization of Reaction Temperature and Time for Basic MIRA Assay

**Figure 2:**
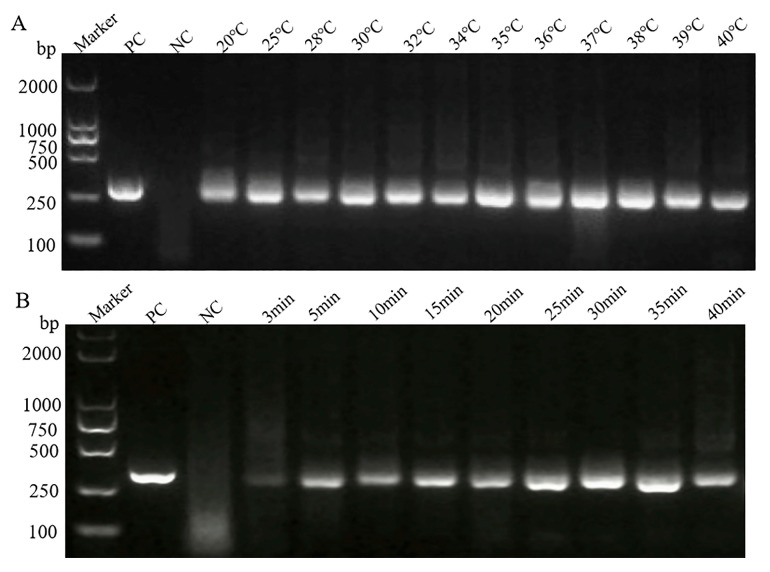
The results of basic MIRA reaction optimization. PC, positive control; NC, negative control. (**A**) MIRA at different temperatures. (**B**) MIRA at different times.

On the basis of determining the better primers, the optimal reaction temperature and reaction time were determined. As shown in Figure 2A, the target bands were generated between 20 °C and 40 °C, and the bands were brightest at 35 °C. A bright band appeared after 5 min, and its intensity peaked at 30 min (Figure 2B). Therefore, 35 °C was selected as the best reaction temperature for the MIRA experiment, and 30 min was the optimal reaction time.

### 3.3. Specificity and Sensitivity

**Figure 3:**
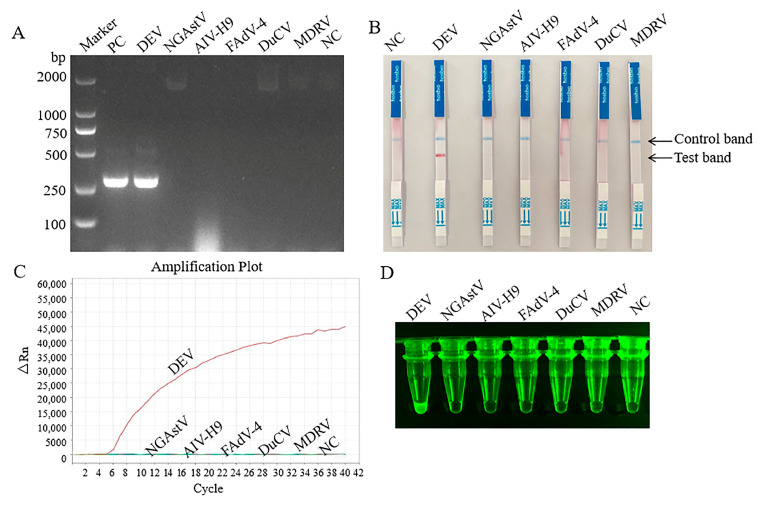
Analytical specificity results of three MIRA assay. PC, positive control; NC, negative control. (**A**) Specificity of basic MIRA for the detection. (**B**) Specificity of MIRA-LFD for the detection. (**C**) Specificity of MIRA-qPCR for the detection. (**D**) Fluorescence imaging of MIRA-qPCR specificity.

Six viruses (DEV, FAdV-4, AIV H9, MDRV, NGAstV, and DuCV) were used to validate the specificity of the basic MIRA, MIRA–qPCR, and MIRA–LFD for DEV. As shown in Figure 3A–D, all three MIRA assays amplified the sequence of DEV, with no cross-reactivity with other pathogens. The negative controls were not amplified. Therefore, all three methods showed good specificity.

**Figure 4:**
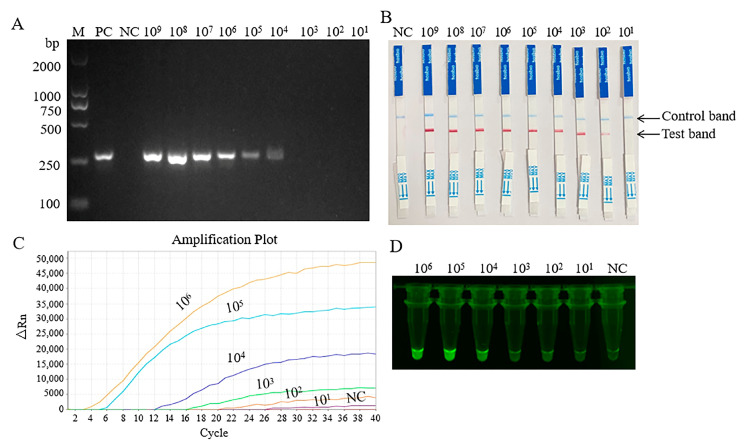
Analytical sensitivity results of three MIRA assay. PC, positive control; NC, negative control; DNA template with 1 × 10^9^ to 1 × 10^1^ copies/μL. (**A**) Sensitivity of basic MIRA for the detection. (**B**) Sensitivity of MIRA-LFD for the detection. (**C**) Sensitivity of MIRA-qPCR for the detection. (**D**) Fluorescence imaging of MIRA-qPCR sensitivity.

To determine the sensitivity of the MIRA, the plasmid template was serially diluted. As shown in Figure 4A–D, the minimum detectable concentration of the basic MIRA was 1 × 10^4^ copies/μL. The detection limits of both MIRA–LFD and MIRA–qPCR were 1 × 10^1^ copies/μL, but the test lines of 1 × 10^2^ copies/μL were relatively clear.

### 3.4. Evaluation of Clinical Samples

To evaluate the usefulness of basic MIRA, MIRA–qPCR and MIRA–LFD assays, 40 clinical samples with suspected DEV infections were tested. Among the samples, eight were positive for DEV, and the positive detection rates of the three MIRA and PCR methods were all 20%. The PCR products of the positive samples were sequenced by Sangon Biotech (Shanghai) Co., Ltd. (China), and all were confirmed positive for DEV. The PCR results were 100% consistent with the MIRA, MIRA–qPCR, and MIRA–LFD results, indicating that MIRA is a reliable tool for the detection of DEV in clinical samples.

## 4. Discussion

DEV is a pathogen that can cause infectious diseases in ducks. It can also cause significant harm to waterfowl such as geese and swans, leading to serious economic losses of waterfowl industry [16]. Therefore, rapid and accurate diagnosis is crucial for the prevention and control of the pathogen. Compared with other animal pathogens, there are not many new methods reported for the detection of DEV. The enzyme-linked immunosorbent assay (ELISA) was a commonly used serological method, mainly used to detect antibodies against DEV in duck serum, which can assist in determining whether ducks are infected with DEV [17]. However, the ELISA method is complex to operate and usually cannot further distinguish the genotypes of pathogens. Using monoclonal antibodies, a colloidal gold-based immunochromatographic assay (ICA) was established, and DEV antigen could be rapidly detected within 15 min, but the consistency of the detection rate of fecal swab samples with PCR was only 80% [18].

PCR is a commonly used molecular biology method that detects viruses by amplifying the specific gene fragments. Based on the specific conserved region UL54 of the duck enteritis virus (DEV) gene, specific detection primers were designed, and the established conventional PCR could recognize DEV [15]. The established multi-quantitative polymerase chain reaction (qPCR) assay based on TaqMan probe was specific and sensitive, with a detection limit of 11.6 copies for DEV [19].

Compared with PCR and qPCR temperature-variable cyclic amplification, the techniques based on isothermal amplification have lower requirements for temperature settings. MIRA technology has developed rapidly in recent years. It has many advantages. The detection can be completed within 30 min at 25–42 °C, and without the need for thermal cyclers. The results are presented in a variety of ways [20,21,22]. In this study, three MIRA assays (basic MIRA, MIRA–qPCR, and MIRA–LFD) targeting TK gene of DEV were established. The basic MIRA reaction could be efficiently performed in 30 min at 35 °C, using a pair of primers. There were no cross-reactions with several common pathogens of waterfowl. The reaction optimization test showed that the target band could be detected at 20 °C, indicating that this method obviously had no strict temperature requirements. Meanwhile, the results of basic MIRA could be initially observed as soon as 5 min, which was also the difference between MIRA and other detection methods such as PCR, qPCR and LAMP.

Detection with MIRA–qPCR and MIRA–LFD was completed within 20 min, and the limit of detection of the two MIRA assays (1 × 10^1^ copies/μL) showed that they were more sensitive than the basic MIRA. This sensitivity was similar to that of the qPCR reported [19]. In addition, the results could also be observed through fluorescence imaging equipment, with consistent specificity and sensitivity. The test results of clinical samples further confirmed the practicality of the MIRA method. Compared with the traditional PCR, the MIRA methods are more sensitive, convenient, and suitable for the rapid detection of DEV.

MIRA technology has demonstrated significant advantages in the detection of various pathogens and genes. However, MIRA is not without limitations, just like other diagnostic techniques. Our experiments revealed that MIRA could be performed with just one primer pair, but the design specifications were highly stringent, particularly for primer length and target gene size. It was recommended to use primers with a length of 28–35 bp. If the primers were too short, it could affect the amplification speed and the specificity of the detection. Amplification efficiency peaked with target fragments ranging from 150 to 300 bp, whereas it decreased with longer fragments. In addition, MIRA–qPCR and MIRA–LFD exhibited higher sensitivity, but false positives were likely to occur under elevated reaction temperatures.

## 5. Conclusions

The basic MIRA, MIRA–qPCR and MIRA–LFD assays were established to detect DEV in this study. All three MIRA methods showed excellent specificity, only generating specific amplification for DEV without cross-reactivity against five common avian viruses (NGAstV, AIV H9, FAdV-4, DuCV, and MDRV). The basic MIRA assay demonstrated a detection limit of 1 × 10^4^ copies/μL. Both MIRA-qPCR and MIRA-LFD exhibited significantly improved sensitivity (1 × 10^1^ copies/μL), representing a 1000-fold increase compared to the basic method. Clinical evaluation of 40 samples revealed that the positive detection rates of all three MIRA methods were fully consistent with conventional PCR, demonstrating the clinical applicability of the three MIRA tests. MIRA technology, with its advantages of simple instrument requirements, short reaction times, and visual reading of test strips, has become a reliable tool for rapid screening of DEV. The MIRA methods developed in this study have significantly improved DEV monitoring efficiency in waterfowl farming.

## Figures and Tables

**Table 1:** Sequences of the primers and probes.

Method	Primer/Probe	Primer Sequences (5′–3′)	Products (bp)
Basic MIRA	F1	ACTGTTGTCGGAGGATACCCTTGCAGCCACT	278
	R1	GCCGATCAATTATGAGCGTAATATCTGGT	
	F2	GCATGTCTTTGCTTCCCAGCAGCTCGTTT	294
	R2	CCAATCTTTGTCCCATTTATCACAGTCCC	
	F3	CCGCATGTCTTTGCTTCCCAGCAGCTCGTT	293
	R3	ATCTTTGTCCCATTTATCACAGTCCCACA	
	F4	AGAAACAACACCACCAGATATTACGCTCAT	345
	R4	CAATCTTTGTCCCATTTATCACAGTCCC	
MIRA-qPCR	F1	ACTGTTGTCGGAGGATACCCTTGCAGCCACT	
	R1	GCCGATCAATTATGAGCGTAATATCTGGT	
	F1-P	TGCCGAGCCAATGGCGTATTGGAGAAATCA[FAM-dT]T[THF][BHQ-dT]GAAGATGTAATAAAG-[C3spacer]	
MIRA-LFD	F1	ACTGTTGTCGGAGGATACCCTTGCAGCCACT	
	[biotin]-R1	[biotin]-GCCGATCAATTATGAGCGTAATATCTGGT	
	F2-P	[FAM]-CCGAGCCAATGGCGTATTGGAGAAATCATT[THF]TGAAGATGTAATAAA-[C3spacer]	

## Data Availability

The original contributions presented in this study are included in the article. Further inquiries can be directed to the corresponding author.
